# Radiocarbon analysis of modern olive wood raises doubts concerning a crucial piece of evidence in dating the Santorini eruption

**DOI:** 10.1038/s41598-018-29392-9

**Published:** 2018-08-09

**Authors:** Yael Ehrlich, Lior Regev, Elisabetta Boaretto

**Affiliations:** D-REAMS Radiocarbon Laboratory, Kimmel Center for Archaeological Science, Scientific Archaeology Unit, Weizmann Institute of Science, Rehovot, 7610001 Israel

## Abstract

Charred olive wood is abundant in the archaeological record, especially around the Mediterranean. As the outermost ring closest to the bark is assumed to represent the latest time that the tree was alive, the radiocarbon date obtained from the outermost rings of an olive branch buried during the Santorini volcanic eruption is regarded as crucial evidence for the date of this cataclysmic event. The date of this eruption has far reaching consequences in the archaeology of the Aegean, Egypt and the Levant, and the understanding of their interconnections. We analyzed the radiocarbon concentrations in cross-sections from a modern olive tree trunk as well as from a living branch, and obtained near-annual resolution dates using the radiocarbon “bomb peak”. In both cases we show that radiocarbon dates of the last formed wood along the circumference are not chronologically homogenous, and can differ by up to a few decades. Thus the outermost wood layer does not necessarily represent the date of the last year of growth. These findings challenge the interpretation of the results obtained from dating the olive branch from the Santorini volcanic eruption, as it could predate the eruption by a few decades. In addition, our results are also significant for any future studies based on archaeologically preserved olive wood.

## Introduction

One important motivation for investigating whether annual growth rings in olive wood (*Olea europaea*) are present and can be successfully identified in olive wood arose recently^1,2^, as an olive branch was used in dating the colossal volcanic eruption of Santorini^3,4^. This dating was based on the assumption that the outermost ring was formed just prior to the eruption. The eruption of Santorini (Thera) was one of the largest volcanic events in historical times^5^, and is a key event in anchoring various archaeological chronologies across the Mediterranean^6–13^. The date of the eruption of Santorini, and therefore too, the dates of archaeological levels that contain the volcanic debris^14^ in the Aegean, as well as in Egypt and the Levant, has been debated for decades.

Based on archaeological correlations between the Aegean, Egypt and the Levant, the eruption of Santorini was believed to have occurred around 1500 BCE, after the beginning of the New Kingdom in Egypt^6,7,12,13^. It should be noted that the beginning of the New Kingdom has been dated by radiocarbon and then modelled to between 1570-1544 BCE^15^.

Radiocarbon dating of short lived organic materials from numerous sites in the Aegean that were derived from the Santorini volcanic context^16–21^ consistently produce dates which are a century earlier than the “archaeological” chronology. For example, a weighted average of 16 measurements by Bronk Ramsey *et al*.^20^ resulted in an uncalibrated radiocarbon age of 3350 ± 10 BP, which when calibrated using the most recent calibration curve^22^ translates to between 1683–1617 BCE (2σ range). This is inconsistent with a date during the 16^th^ century BCE.

Due to the nature of the calibration curve during this time period, it has been argued that there is a small probability that the radiocarbon age of 3350 BP may be calibrated to the 16^th^ century^7^. The calibrated date ranges of the majority of short lived organic material indeed indicate a small probability of having originated in the 16^th^ century BCE, although the most probable range lies in the 17^th^ century BCE^16^. The key evidence for dating the eruption exclusively to the 17^th^ century is the charred wood from an olive branch found buried under the tephra at Santorini. The assumption is that the latest wood cells formed near the bark represents the last year of growth, before being buried under the tephra, and the wood growth layers further away from the bark are older. Thus a sequence of chronologically consecutive samples may be “wiggled-matched” to find an exact location on the radiocarbon calibration curve^4^. The resulting calibrated date range for the latest wood sample near the bark, using the latest IntCal13^22^, is 1656–1609 BCE (2σ range) or 1632-1615 (1σ range). Based on this analysis, the latest wood was not deposited in the 16^th^ Century. In this study we addressed the question of whether or not the outermost ring in olive trees is indeed produced just prior to the death of the tree.

Identifying annual rings in olive wood is not straightforward^1^. Polished olive wood sections do show what appear to be rings, however it is known that intra-annual density fluctuations (IADFs) may be visually mistaken as true rings^1,23^. Furthermore, asymmetric cambial activity may cause discontinuities along growth rings, as little or no xylem is produced in areas of reduced cambial activity^24^. This irregular cambial activity around the circumference of the tree is responsible for the characteristic non-circular cross section of olive trees^25^. Based on radiocarbon concentrations, we reported^26^ that visually identified growth rings in olive trees are not necessarily annual, as there is a discrepancy between the number of rings identified and the number of years expected between them. We therefore try to avoid using ring number terminology, and refer to sampling points using arbitrary numbers for each cross-section. Specifically, the samples collected around the circumference should not be considered as originating from the last ring *per se*, rather to mean they have been collected from nearest to the bark as was possible.

The olive tree trunk is composed of independent vascular systems, which results in a sectorial interaction between major roots and specific sections of the crown^25^. Therefore, when certain roots are damaged, the specific section of the crown relying on that water supply will die. Cambial growth in the respective vascular region in the stem will also cease, while the rest of the tree can continue to live normally^25^. A similar phenomenon, where regions on the circumference of the tree’s cross-section may die while others continue to grow have been reported for a number of coniferous species growing under adverse conditions^27–29^.

As part of the olive tree’s normal growth, new trunks may develop around the base of the original tree^25^, and as they grow in close proximity, the friction at their interface can cause bark breakage. The wearing of the bark can expose live parenchyma cells, which can create “tissue bridges” eventually forming continuous cambium tissue^30^. This process may result in wood of different ages from independent trunks or branches to merge and become visually undistinguishable.

In this work, we explore the wood of modern olive trees grown in northern Israel, utilizing radiocarbon dating at near-annual resolution, enabled by the “bomb peak”^31^. Due to atmospheric nuclear experimentation beginning in the late 1950s, atmospheric ^14^C levels nearly doubled over a time span of merely one decade, and were in turn imprinted in organic material due to the short carbon cycle. After these experiments were internationally banned, ^14^C levels gradually decreased^32^. This sudden increase and subsequent gradual decrease is referred to as the “bomb peak” or “bomb pulse”. The dramatic change in ^14^C over a short period of time enables assigning a certain ^14^C concentration to a very narrow window of time, often within one or two years. This is in contrast to calibrating radiocarbon dates from earlier times, where the changes in ^14^C were much more subtle, leading to the assignment of a wider window of time possible for dating the sample.

Here we examine a cross-section from a living olive branch bearing green leaves, and we expand the investigation of the whole olive tree cross-section studied previously by us^26^. We first determine their chronological growth deposition using high resolution radiocarbon dating. We then discuss the consequences of our observations to dating the Santorini eruption and the broader implications for archaeology.

## Results

**Figure 1:**
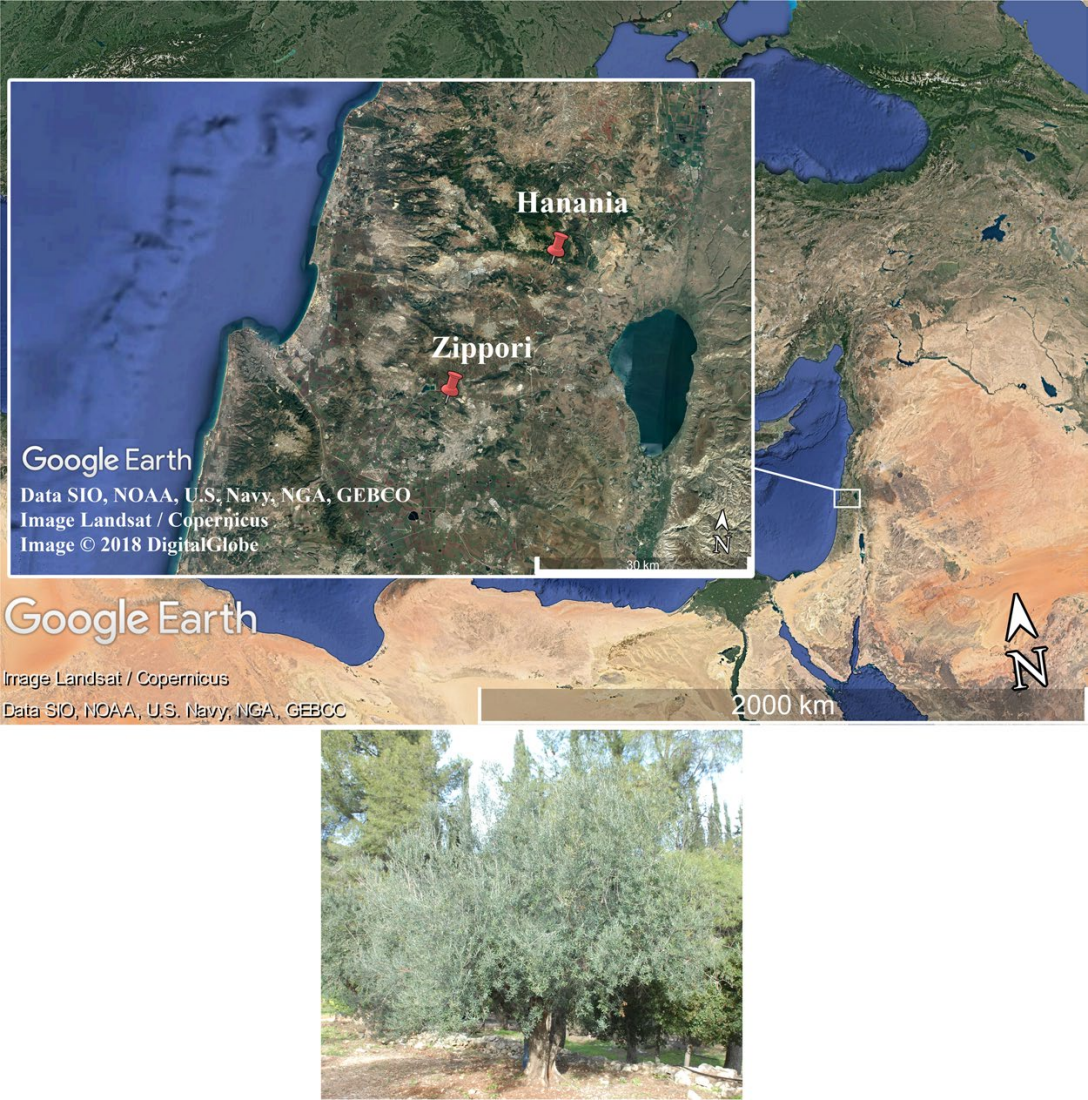
Top: Map of the sampling region in northern Israel (inset, scale bar of 30 km), within larger map of the Mediterranean (scale bar of 2000 km). Bottom: live olive tree sampled at Havat Hanania (northern point in the inset map) is shown in the center of the photo. Maps generated with Google Earth.

We first analyzed a living branch, which was bearing other smaller branches with many green leaves from an olive tree growing at a location called Havat Hanania in northern Israel (Fig. 1). The branch was cut in 2013, and the tree was likely originally planted in the 1930s.

### Havat Hanania- cross-section of a live branch

**Figure 2:**
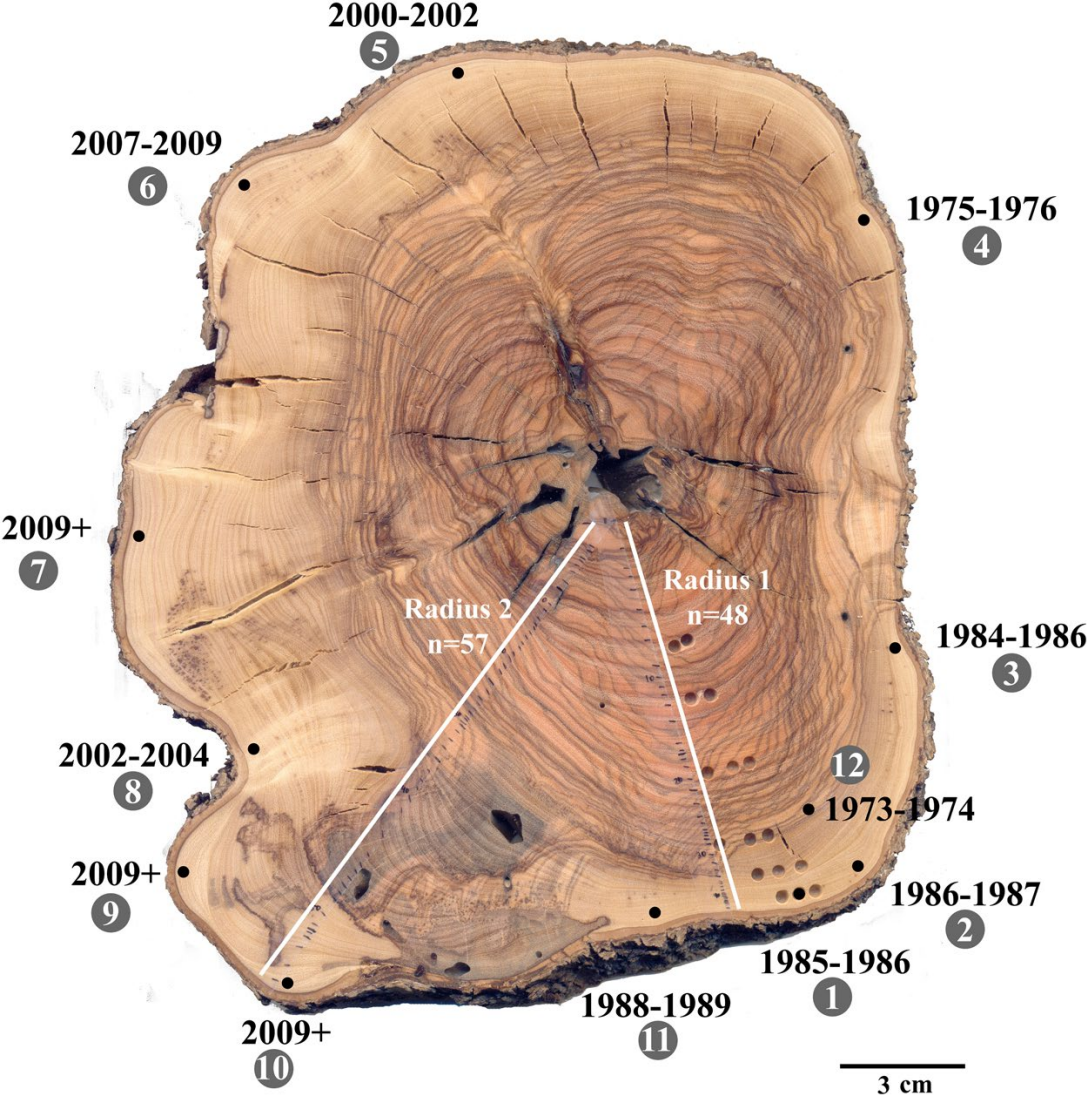
Cross section of olive tree branch cut down alive in 2013, in Havat Hanania in northern Israel, with sampling points for radiocarbon marked with black dots around the circumference. Sample numbers (white font on gray circle) and resulting calibrated calendar years are indicated on the outer perimeter.

The branch sampled from Havat Hanania (Fig. 2) was cut down alive, and many branches bearing green leaves were growing from it. Thus the outermost ring is expected to date to around 2013, the year the branch was cut. It should be noted that the latest bomb ^14^C calibration curve, NHZ2^32^, enables calibration up until the end of 2009. Thus, we refer to samples which have lower ^14^C concentrations than those characteristic of 2009, and which are clearly post-2009 (near the bark), as 2009+.

Table 1 presents the dating results of this section. Only 3 of the 11 wood samples from just beneath the bark gave a 2009+ date, while all the others were older. Four of the dates are in the 1980s (points 1–3 and 11 in Fig. 2) and the earliest sample is from 1975–1976 (point 4 in Fig. 2). There is thus a high probability that the age of the wood just below the bark does not represent the time when the branch was cut and the discrepancy can be as much as 40 years.

**Table 1:** F^14^C of samples from olive branch cross section from Havat Hanania (Fig. 2).

Sample number on figure	Lab No.	F^14^C	F^14^C+/−	Calibrated 1σ range (2σ in bold)	Likely age based on radiocarbon and location in wood
1	7692	1.20034	0.00378	1985–1986 (68.2%)	1985–1986
2	8635	1.19279	0.00318	1986–1987 (68.2%)	1986–1987
3	8575	1.20500	0.00304	1984–1986 (68.2%)	1984–1986
4	8576	1.36878	0.00307	1975–1976 (68.2%)	1975–1976
5	8304	1.08556	0.00411	2000–2002 (68.2%)	2000–2002
6	7876	1.05541	0.00206	1956–1957 (8.4%) 2007–2009 (59.8%)	2007–2009
7	8574	1.03647	0.00281	1956–1956 (68.2%) **1956–1956 (92.8%)** **2009–2009 (2.6%)**	Post-2009
8	8206	1.07632	0.00214	2002–2004 (68.2%)	2002–2004
9	7875	1.03773	0.00215	1956–1956 (68.2%) **1956–1956 (89.0%)** **2008–2008 (1.4%)** **2009–2009 (5%)**	Post-2009
10	8303	1.04067	0.00385	1956–1956 (68.2%) **1956–1957 (62.1%)** **2008–2009 (33.3%)**	Post-2009
11	8205	1.17272	0.00312	1988–1989 (68.2%)	1988–1989
12	8634	1.43358	0.00404	1962–1962 (5.2%) 1973–1974 (63.0%)	1973–1974

Calibrated dates according to OxCal^38^ are presented based on the NHZ2 calibration curve^32^. All calibrated dates are within the 1σ range, probabilities are presented as percentages. For dates beyond the current calibration curve (after 2009), 2σ range is shown and indicated in bold. From the possible date ranges suggested by radiocarbon, we present our subjective likely option in the last column, based on the radiocarbon probability, as well as the location of the sample (near the bark are expected to be ~2013, for which the calibration curve is not yet updated and instead provides an output of ~1956).

It should be noted that for radius 1, fewer rings were identified than for radius 2. The difference in the number of rings identified between radii (57−48 = 9) does not match the two decades of difference in years obtained for the last rings of these radii. Assuming the tree was indeed planted in the 1930s, the 48^th^ ring of radius 1 would indeed be expected to date to the 1980s. However, for radius 2, the 57^th^ ring would be expected to date to the 1990s, rather than the resulting date of 2008–2009. Thus for radius 2, our visual ring count has underestimated the true number of years, serving as an additional example of the difficulty of identifying annual rings in olive wood. For sampling results along radius 1, see Table S1.

As more than half the samples from the outermost ring dated to well before 2013, we decided to re-examine the trunk of another olive tree from a different location in northern Israel that was studied previously, Zippori^26^ (Fig. 1).

### Zippori- entire trunk cross section, dead tree

**Figure 3:**
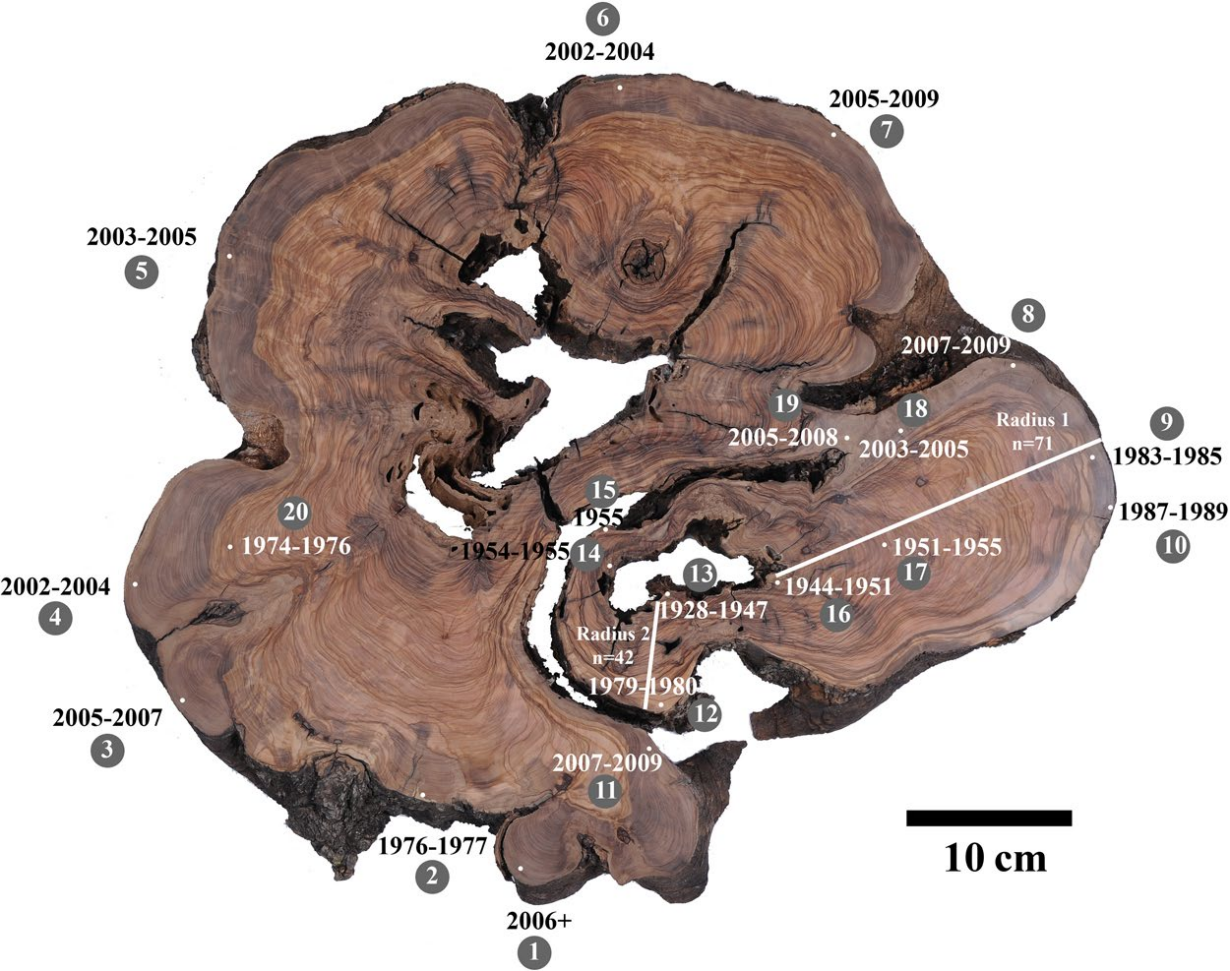
Transverse section of olive tree trunk, cut down in 2013 in Zippori, northern Israel, with sampling points for radiocarbon marked on cross section with small white dots (to scale of the 2.8 mm samples). Near each dot is the sample number (white font on gray circle) and the calibrated calendar years. Two radii are indicated with white lines along the sub-section to the bottom right of the figure, with the number of growth rings identified indicated (radius 1 n = 71; radius 2 n = 42). © Weizmann Institute of Science. Original olive section photo by: Itai Belson.

In total, 20 samples (14 new samples compared to the previous study^26^) were analyzed for radiocarbon dating from a cross-section of a whole olive tree cut down a few years after the tree had died, from the site of Zippori in northern Israel. As the tree had been dead for only a few years before felling in 2013, the wood nearest the bark would be expected to fall within a few years from this date. Indeed, the cellulose extracted from many samples of the outermost wood were dated in the range of 2002–2009 (points 1, 3–8 in Fig. 3, and Table 2). However in the segment that includes radii 1 and 2 (see Fig. 3), 5 analyses of wood below the bark (points 9,10,12, 14 and 15) produced older dates, with the oldest being around 1955. This segment appears to belong to a separate branch within the trunk (see also Table 2). Interestingly, points 18 and 19, from the same segment but in an area where no growth patterns are observed, are both dated to the 2000s (Fig. 3 and Table 2). Thus the dates from the wood beneath the bark of this segment range from 1955 to the time of felling.

**Table 2:** F^14^C of samples from olive wood cross section from Zippori (Fig. 3).

Sample number on figure	Lab No.	F^14^C	F^14^C +/−	Calibrated 1σ range (2σ)	Likely age based on radiocarbon and location in wood
1	7337	1.05722	0.00489	1956–1957 (7.4%) 2006-… (60.9%)	2006+
2	8600	1.35141	0.00316	1976–1977 (68.2%)	1976–1977
3	7374	1.06118	0.00328	1957–1957 (1.6%) 2005–2007 (66.6%)	2005–2007
4	7334	1.07782	0.00291	2002–2004 (68.2%)	2002–2004
5	7331	1.07259	0.00298	2003–2005 (68.2%)	2003–2005
6	8577	1.07702	0.00286	2002–2004 (68.2%)	2002–2004
7	7379	1.05895	0.00583	1957–1957 (6.2%) 2005–2009 (62.1%)	2005–2009
8	8592	1.05514	0.00267	1956–1957 (8.6%) 2007–2009 (59.6%)	2007–2009
9	7383	1.21499	0.00568	1960–1961 (23.1%) 1983–1985 (45.1%)	1983–1985
10	8637	1.17505	0.00429	1987–1989 (68.2%)	1987–1989
11	8599	1.05475	0.00276	1956–1957 (9.0%) 2007–2009 (59.3%)	2007–2009
12	7342	1.28579	0.00353	1962–1962 (13.1%) 1979–1980 (55.1%)	1979–1980
13	7339	0.97936	0.00341	1668–1685 (11.3%) 1732–1781 (35.5%) 1798–1807 (6.6%) 1928–1947 (12.9%) 1951–1954 (1.9%)	1928–1947
14	8597	0.99397	0.00261	1711–1717 (5.8%) 1828–1831 (1.9%) 1891–1909 (57.9%) 1954–1955 (2.6%)	1954–1955
15	8596	1.01226	0.00264	1955–1955 (68.2%)	1955
16	7340	0.97331	0.00303	1651–1669 (27.9%) 1780–1798 (32.2%) 1944–1951 (8.1%)	1944–1951
17	7382	0.98601	0.00489	1688–1730 (19.5%) 1809–1892 (39.9%) 1907–1926 (8.8%)	1951–1955
				**1677–1766 (32.4%)** **1771–1777 (1.1%)** **1800–1941 (61.0%)** **1951–1955 (0.8%)**	
18	8593	1.07308	0.00265	2003–2005 (68.2%)	2003–2005
19	7381	1.06292	0.00517	1957–1957 (4.2%) 2005–2007 (61.4%) 2008–2008 (2.6%)	2005–2008
20	7335	1.38099	0.00683	1974–1976 (68.2%)	1974–1976

Calibrated dates according to OxCal^38^ based on the NHZ2 calibration curve^32^. All calibrated dates are within the 1σ range, probabilities are presented as percentages. Where the 2σ range is shown, it is presented in red. From the possible date ranges suggested by radiocarbon, we present our subjective likely option in the last column, based on the radiocarbon probability, as well as the location of the sample (e.g. consecutive samples must be in chronological order, correlation to estimated ring count and ring growth patterns).

The number of growth rings identified on radius 2 (n = 42) is in agreement with the difference in years between point 13 (1928–1954) and point 12 (1979–1980), although the range is quite large. However, for radius 1 (n = 71) the difference in years between point 16 (1944–1952) and point 9 (1983–1985) is too small compared with the number of rings identified, suggesting false rings were counted. In addition, point 14 on the inner part of this segment had a broad possible dating range of 1890–1955, while point 15, near the bark was dated to 1955.

Point 2 from another segment of the trunk produced an age in the 1970′s, even though it too was sampled beneath the bark, (Fig. 3; Table 2). Following the putative growth ring patterns, it seems point 2 can be traced to point 20, which was likewise dated to the 1970s.

### Ruling out artifact sampling

Samples were collected as sawdust from the wood section using a 2.8 mm drill bit. Visually, the 2.8 mm samples did not appear to span more than a few rings. However, as annual rings in olive wood are difficult to identify, the possibility of an artifact date caused by accidentally sampling a large number of rings in one sample might be possible. Considering this, the earlier than expected dates obtained for numerous points sampled beneath the bark, might possibly have been caused by the 2.8 mm diameter spanning numerous older unidentified rings, which would lead to higher concentrations of ^14^C, and in turn, an earlier date. Theoretical considerations why this is unlikely are in the supplementary material.

In order to empirically rule out the possibility that the obtained date beneath the bark is a result of unintentional sampling of a larger span of years, a microtome was used to sample thin sections very close to the bark and compare to thin sections approximately 1 cm inwards toward the pith. Microtome thin sections were cut from the Zippori whole tree cross-section, as close to the bark as possible, in an area assumed to represent the last year of growth of this section, as well as from expectedly slightly earlier wood, approximately 1 cm toward the pith. The sample closest to the bark was dated to 1987–1989 (point 10), while the sample collected ~1 cm toward the pith from point 10 was dated to 1984–1985 (see Table 2). Thus, the initial result of point 9 dating the last year of growth of this subsection to 1985 at the latest is reliable to within a few years. Thus the last year of growth of this subsection clearly differs from the other points around the circumference of the rest of the trunk which were dated to the 2000s.

For the section from the Havat Hanania branch (Fig. 2), the sample closest to the bark (point 2) was dated to 1985–1987, while the sample slightly inwards towards the pith was dated to 1973–1974 (Fig. 2). This is in agreement with the rest of the points dated in the vicinity (points 1, 3, 11 in Fig. 2), which were sampled as sawdust collected after drilling with a 2.8 mm drill bit.

## Discussion

The interest in the identification of annual growth rings in olive wood was reignited^1^, following the use of an olive branch to date the eruption of Santorini^3^. Our study identifies another possible error which stems from the assumption that the outermost part of the olive wood, closest to the bark, is contemporaneous with the last year of growth. Thus, dating the outermost wood of an olive tree buried alive by some catastrophic event, may not necessarily correspond to the year the event occurred, but could produce an apparent date of up to 40–50 years earlier than the time that the tree actually died. With respect to dating Santorini, our results raise doubts about the reliability of the date obtained from the olive branch. This date is widely regarded as a crucial piece of evidence for dating this important eruption. However, it should be noted that the short lived organic material radiocarbon dates of the Santorini eruption^16–20^ still constitute good evidence that the eruption occurred in the 17^th^ century BCE.

We have shown here and previously^26^ that the visual identification of growth rings in olive wood is unreliable. This is consistent with previous reports^1,33^, but here we use the tool of radiocarbon dating at high resolution, enabled by the “bomb peak” to demonstrate this. We have shown that in order to either verify or rule out the possibility of olive trees forming annual wood growth, a method more objective and precise than visual identification would be required. The utilization of the “bomb peak” can aid in the validation of any such method, for wood from ~1955 and onwards.

The compound analyzed for dating in this work was cellulose. The main source of carbon for synthesizing cellulose in plants is phloem-transported sugars, which, in turn, could be a breakdown product of starch^34^. The possibility that old carbon was used for modern growth, causing radiocarbon dating to underestimate the age of the wood is not likely, as there is no mechanism to explain why old carbon, such as starch from previous years, would not be dispersed relatively homogenously around the circumference, or how it could be stored for many years and suddenly be utilized only in one restricted area. In addition, it may be possible for starch to be stored in wood for a few years, but it is unlikely that starch (or any other major source of carbon) would remain in the tree for decades in relative abundance such as to bias the resulting date specifically in one area and not in others. Thus, the most plausible explanation for the lack of homogeneity around the circumference is that the cambium stopped being active at these points, and that this is not an artifact due to utilization of old carbon.

Charred olive wood is fairly common in archaeological sites around the Mediterranean. These charred fragments are potentially of much value for dating and reconstructing past environments and climate. This study clearly shows that olive trees do not systematically produce visible growth rings and that growth cessation is a common phenomenon. Thus any applications using archaeological olive wood would need to take this into account.

## Materials and Methods

### Study area and sampling procedure

Two olive trees (*Olea europeae*) were sampled from northern Israel during 2013 (Fig. 1): one from Zippori (32°45′24.1″N 35°16′50.9″E, 195 m, see also^26^) and the other from Havat Hanania (32°56′15.3″N 35°25′29.6″E, 415 m). The tree in Zippori had died several years prior to cutting it down, and thus an entire cross-section of the trunk was obtained (Fig. S3). The tree in Havat Hanania was alive and a cross-section of a branch was collected during routine tree maintenance trimming. The trees at Havat Hanania are thought to have been planted during the British mandate in the 1930s. Both wood samples were polished gradually to 1000 grit grade. The olive transverse wood sections required a belt sander (Makita #9404) with five grades of grit (24, 40, 80, 150, 320) followed by polishing with a random orbit sander (Makita #BO5041) with four grades of grit (400, 600, 800 and 1000). Samples for α-cellulose extraction were obtained using a Dremel drill (8000 model, using a 2.8 mm drill bit), collecting the accumulated saw dust at each point. The maximum drilling depth into the wood was approximately 5 mm, which required drilling of 2–3 holes per sample. The cross-sections were finely polished (as described above), and it was therefore possible to easily eliminate sawdust from the surface of the section using Kimwipes® and pressurized air between sample collection. In addition, between samples the drill bit was cleaned with moist Kimwipes® (using double distilled water) and dried with pressurized air. As an additional sampling method, ~30 mg of wood were collected by pooling thin sections cut from a sampling region, using a WSL-Lab Microtome^35^. These samples also underwent α-cellulose extraction for subsequent radiocarbon dating.

### Dendrochronological methods

Tree rings were visually identified under a binocular microscope (M80, Leica) and measured to the nearest 0.001 mm using a sliding micrometer stage (“TA” measurement system, Velmex Inc.) and the Tellervo dendrochronological analysis package^36^.

### α-cellulose extraction

Wood sawdust was placed in 16 × 125 mm borosilicate glass test tubes. All glassware, including tubes and Pasteur pipettes, was pre-baked (1 hour at 450 °C) to eliminate organic contamination. The tops of the tubes were stuffed with meshed glass wool. Acid-base-acid (ABA) pretreatment was carried out: samples were treated with aliquots of 5 ml 1 N HCl for 1 hour; washed with DDW; treated with 5 ml of 0.1 N NaOH for 1 hour; washed with DDW and finally treated with 5 ml 1 N HCl for 1 hour in a water bath at 70 °C to remove any carbon that may have adhered during the previous alkaline treatment. After washing with DDW, holocellulose was extracted using a modification to a variation suggested by Southon and Magana (2010) of the Jayme-Wise method: 2.5 ml of 1 N HCl and 2.5 ml of 1 M NaClO_2_ were added to the samples which were then transferred to a water bath at 70 °C and left overnight. For samples which required further bleaching, the treatment in 2.5 ml of 1 N HCl and 2.5 ml of 1 M NaClO_2_ was repeated until all samples became white. After bleaching, the samples were washed with DDW. For the extraction of α-cellulose, the samples were treated with 6 ml of 5 N NaOH for 1 hour, followed by washing with DDW and subsequently treated for 1 hour with 5 ml of 1 N HCl in a water bath at 70 °C. The samples were then washed with DDW until reaching a neutral pH, and dried in an oven at 100 °C.

### Radiocarbon dating

Between 2–4 mg of α-cellulose were weighed into pre-baked (1 hour at 900 °C) quartz tubes containing 200 mg CuO and oxidized to CO_2_ in a vacuum line at 900 °C for 3 hours. CO_2_ pressure known to result in ~1 mg carbon was transferred from each sample into tubes containing 1 mg of activated Co for graphitization. ^14^C content determination on the resulting graphite was carried out at the Dangoor Research Accelerator Mass Spectrometry (D-REAMS) laboratory at the Weizmann Institute. All calculated ^14^C ages were corrected for isotopic fractionation based on the stable carbon isotope ratio (δ^13^C value, as measured by the AMS). Calibrated ages in calendar years were obtained from the NHZ2 calibration curve^32^ using OxCal v 4.2^37^.

## Electronic supplementary material

Supplementary Information

## References

[1] Cherubini P (2013). Olive Tree-Ring Problematic Dating: A Comparative Analysis on Santorini (Greece). PLoS One.

[2] Cherubini P (2014). The olive-branch dating of the Santorini eruption. Antiquity.

[3] Friedrich WL (2006). Santorini eruption radiocarbon dated to 1627-1600 BC. Science.

[4] Friedrich WL (2014). The olive branch chronology stands irrespective of tree-ring counting. Antiquity.

[5] Sigurdsson H (2006). Marine Investigations of Greece’s Santorini Volcanic Field. EOS, Trans. Am. Geophys. Union.

[6] Bietak M (2015). Recent Discussions about the Chronology of the Middle and the Late Bronze Ages in the Eastern Mediterranean: Part I. Bibl. Orient..

[7] Wiener, M. H. Dating the Theran Eruption: Archaeological Science Versus Nonsense Science. In Israel′s Exodus in Transdisciplinary Perspective (ed. Levy, T. E.) 131–143, 10.1007/978-3-319-04768-3, (Springer International Publishing, 2015).

[8] Höflmayer F (2012). The date of the Minoan Santorini eruption: quantifying the “offset”. Radiocarbon.

[9] Polinger Foster, K., Sterba, J. H., Steinhauser, G. & Bichler, M. The Thera eruption and Egypt: pumice, texts, and chronology. In *Time’s Up! Dating the Minoan Eruption of Santorini: Acts of the Minoan Eruption Chronology Workshop*, *Sandbjerg November 2007* (ed. Warburton, D. A.) 171–180 (2009).

[10] Doumas, C. G. *Thera: Pompeii of the ancient Aegean*. (Thames and Hudson, 1983).

[11] MacGillivray, J. A. T, Hatshepsut, and the Keftiu: Crisis and Response in Egypt and the Aegean in the Mid-Second Millennium BC. In *Time’s Up! Dating the Minoan Eruption of Santorini: Acts of the Minoan Eruption Chronology Workshop*, *Sandbjerg November 2007* (ed. Warburton, D. A.) 154–170 (2009).

[12] Warren, P. The date of the Late Bronze Age eruption of Santorini. In *Time’s Up! Dating the Minoan Eruption of Santorini: Acts of the Minoan Eruption Chronology Workshop*, *Sandbjerg November 2007* (ed. Warburton, D. A.) 181–186 (2009).

[13] Höflmayer, F. Aegean-Egyptian synchronisms and radiocarbon chronology. In *Time’s Up! Dating the Minoan Eruption of Santorini: Acts of the Minoan Eruption Chronology Workshop*, *Sandbjerg November 2007* (ed. Warburton, D. A.) 187–195 (2009).

[14] Huber H, Bichler M, Musilek A (2003). Identification of Pumice and Volcanic Ash from Archaeological Sites in the Eastern Mediterranean Region Using Chemical Fingerprinting. Egypt and the Levant.

[15] Ramsey CB (2010). Radiocarbon-based chronology for dynastic Egypt. Science.

[16] Manning SW (2014). Dating the Thera (Santorini) eruption: archaeological and scientific evidence supporting a high chronology. Antiquity.

[17] Panagiotakopulu E, Higham T, Sarpaki A, Buckland P, Doumas C (2013). Ancient pests: The season of the Santorini Minoan volcanic eruption and a date from insect chitin. Naturwissenschaften.

[18] Bruins HJ (2008). Geoarchaeological tsunami deposits at Palaikastro (Crete) and the Late Minoan IA eruption of Santorini. J. Archaeol. Sci..

[19] Bruins HJ, van der Plitch J, MacGillivray JA (2009). The Minoan Santorini eruption and tsunami deposits in Palaikastro (Crete): Dating by geology, archaeology, ^14^C, and Egyptian chronology. Radiocarbon.

[20] Bronk Ramsey C, Manning SW, Galimberti M (2004). Dating the volcanic eruption at Thera. Radiocarbon.

[21] Wild EM (2010). ^14^C dating of the Early to Late Bronze Age stratigraphic sequence of Aegina Kolonna, Greece. Nucl. Instruments Methods Phys. Res. Sect. B Beam Interact. with Mater. Atoms.

[22] Reimer PJ (2013). IntCal13 and Marine13 Radiocarbon Age Calibration Curves 0–50,000 Years cal BP. Radiocarbon.

[23] Battipaglia G (2016). Structure and Function of Intra–Annual Density Fluctuations: Mind the Gaps. Front. Plant Sci..

[24] Almero G, Terral JF, Arnold-Simard G (1996). Beginnings of Olive Cultivation in Eastern Spain in Relation to Holocene BioclimaticChanges. Quat. Res..

[25] Lavee, S. Biology and physiology of the olive. *World Olive Encyclopaedia* 59–110 (1996).

[26] Ehrlich Y, Regev L, Kerem Z, Boaretto E (2017). Radiocarbon dating of an olive tree cross-section: New insights on growth patterns and implications for age estimation of olive trees. Front. Plant Sci..

[27] Daniels LD, Dobry J, Klinka K, Feller MC (1997). Determining year of death of logs and snags of *Thuja plicata* in southwestern coastal British Columbia. Can. J. For. Res..

[28] Larson D, Matthes-Sears U, Kelly P (1993). Cambial Dieback and Partial Shoot Mortality in Cliff-Face Thuja occidentalis: Evidence for Sectored Radial Architecture. Int. J. Plant Sci..

[29] Cherubini P (2002). Tree-life history prior to death: Two fungal root pathogens affect tree-ring growth differently. J. Ecol..

[30] Schweingruber, F. H. Modification of the Tree-Ring Structure Due to Deformed Stems and Anastomosis. in Wood Structure and Environment (eds Timmel, T. & Wimmer, R.) 231 (2007).

[31] Hua Q, Barbetti M (2004). Review of trophospheric bomb 14C data for carbon cycle modelling and age calibration purposes. Radiocarbon.

[32] Hua Q, Barbetti M, Rakowski AZ (2013). Atmospheric radiocarbon for the period 1950-2010. Radiocarbon.

[33] Cherubini P (2003). Identification, measurement and interpretation of tree rings in woody species from mediterranean climates. Biol. Rev. Camb. Philos. Soc..

[34] Tcherkez G, Ghashghaie J, Griffiths H (2007). Methods for improving the visualization and deconvolution of isotopic signals. Plant, Cell Environ..

[35] Gärtner H, Lucchinetti S, Schweingruber FH (2015). A new sledge microtome to combine wood anatomy and tree-ring ecology. IAWA J..

[36] Brewer PW (2014). Data Management in Dendroarchaeology Using Tellervo. Radiocarbon.

[37] Bronk Ramsey C (2009). Bayesian Analysis of Radiocarbon Dates. Radiocarbon.

[38] Bronk Ramsey C (1995). Radiocarbon Calibration and Analysis of Stratigraphy: the OxCal Program. Radiocarbon.

