# Supplementary material for: Radiocarbon analysis of modern olive wood raises doubts concerning a crucial piece of evidence in dating the Santorini eruption
Source: Sci Rep. 2018 Aug 9;8:11841. doi: 10.1038/s41598-018-29392-9 (PMC6085306; doi:10.1038/s41598-018-29392-9)
Supplement: Supplementary file 1 — Supplementary Information [file 41598_2018_29392_MOESM1_ESM.docx]

**Radiocarbon analysis of modern olive wood raises doubts concerning a crucial piece of evidence in dating the Santorini eruption**

**Yael Ehrlich^1^, Lior Regev^1^, Elisabetta Boaretto^1*^**

^1^ D-REAMS Radiocarbon Laboratory, Kimmel Center for Archaeological Science, Scientific Archaeology Unit, Weizmann Institute of Science, Rehovot, Israel 7610001

**Supplementary material**

**Table S1**

**Figure S1**

**OxCal model**

**Theoretical considerations**

**Figure S3**

**Table S1.** F^14^C values for α-cellulose of samples along radius 1 of the olive wood branch from Havat Hanania. The putative ring or ring range from which the sample was taken is indicated, as well as the unmodelled and modelled calendar year range obtained with Oxcal.

| Sample number | Ring no. | Lab no. | F^14^C | F^14^C +/- | Calibrated 1σ range  unmodelled | Modelled 1σ range Sequence function | Δyears | Δrings | Match between age and ring count |
| --- | --- | --- | --- | --- | --- | --- | --- | --- | --- |
| 1 | 1 | 7871 | 0.97753 | 0.00194 | 1667-1681 (17.0%)  1739-1744 (4.8%)  1763-1783 (24.2%)  1797-1801 (5.4%)  1938-1952 (16.7%) | 1918-1923 (27.1%)  **1945-1952 (41.1%)** |  |  |  |
| 2 | 5 | 7872 | 0.98304 | 0.00278 | 1681-1697 (10.0%)  1725-1738 (7.9%)  1745-1748 (1.2%)  1750-1763 (5.8%)  1802-1815 (7.5%)  1835-1878 (22.0%)  1917-1938 (13.0%)  1952-1953 (0.3%)  1954-1954 (0.4%) | 1920-1925 (25.7%)  **1951-1953 (42.5%)** | 0-8 | 4 | + |
| 3 | 9 | 7873 | 0.99012 | 0.00196 | 1707-1719 (14.2%)  1820-1822 (2.0%)  1826-1832 (7.5%)  1882-1914 (44.3%)  1954-1955 (0.1%) | 1923-1925 (9.9%)  **1952-1953 (58.3%)** | 0-2 | 4 | - |
| 4 | 10 | 7874 | 0.98701 | 0.00193 | 1695-1713 (14.2%)  1715-1726 (8.4%)  1813-1836 (17.7%)  1843-1852 (5.6%)  1868-1873 (3.2%)  1876-1891 (12.0%)  1908-1918 (7.2%) | 1925-1926 (9.5%)  **1952-1954 (58.7%)** | 0-2 | 1 | + |
| 5 | 12 | 7688 | 0.97499 | 0.00326 | 1657-1679 (21.1%)  1764-1773 (6.8%)  1776-1800 (27.3%)  1939-1952 (13.0%) | 1943-1947 (8.0%)  **1953-1954 (60.2%)** | 0-2 | 2 | + |
| 6 | 19 | 7689 | 0.98483 | 0.00349 | 1684-1708 (12.1%)  1718-1732 (7.3%)  1807-1827 (9.5%)  1832-1889 (30.4%)  1911-1928 (8.8%) | **1953-1955 (68.2%)** | 0-2 | 7 | - |
| 7 | 30 | 7690 | 1.41897 | 0.00431 | 1962-1962 (10.6%)  1974-1974 (57.6%) | **1962-1963 (68.2%)** | 7-10 | 11 | - |
| 8 | 33-36 | 7691 | 1.46153 | 0.00434 | 1971-1971 (9.3%)  1972-1973 (58.9%) | 1962-1963 (11.7%)  **1972-1973 (56.5%)** | 9-11 | 3-6 | - |
| 9 | 43-47 | 7692 | 1.20034 | 0.00378 | 1985-1985 (14.0%)  1985-1986 (54.2%) | **1985-1986 (68.2%)** | 12-14 | 7-14 | + |


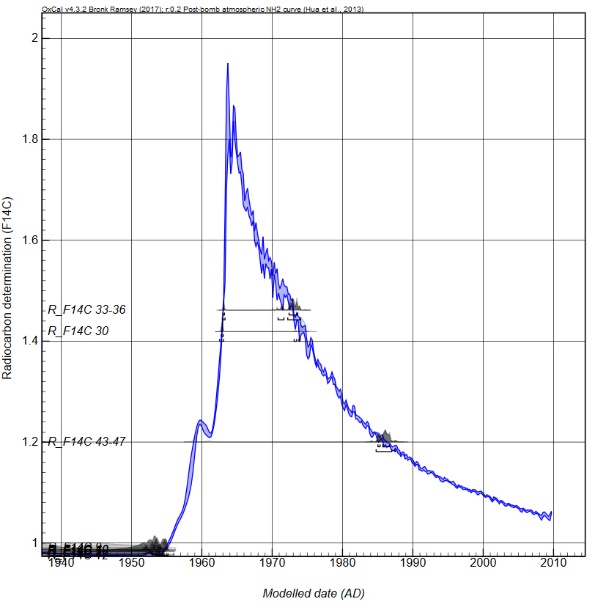


**Figure S 1.** Bomb peak curve with modelled dates indicated (see Table S1).

Sequence model used in Oxcal:

Options()

{

Resolution=0.2;

Curve="Bomb13NH2.14c";

};

Plot()

{

Sequence()

{

Boundary("Start 1");

Sequence("1")

{

After(C_Date("Year",1900,2));

R_F14C("7871", 0.97753, 0.00194);

R_F14C("7872", 0.98304, 0.00278);

R_F14C("7873", 0.99012, 0.00196);

R_F14C("7874", 0.98701, 0.00193);

R_F14C("7688", 0.97499, 0.00326);

R_F14C("7689", 0.98483, 0.00349);

R_F14C("7690", 1.41897, 0.00431);

R_F14C("7691", 1.46153, 0.00434);

R_F14C("7692", 1.20034, 0.00378);

};

Boundary("End 1");

};

};

### Theoretical considerations

Samples were collected as sawdust from the wood section, using a 2.8 mm drill bit. Visually, the 2.8 mm samples did not appear to span more than a few rings. However, as annual rings in olive wood are difficult to identify, the possibility of an artifact date caused by accidentally sampling a large number of rings in one sample might be possible. Considering this, the earlier than expected dates obtained for numerous points, such as 9 and 10 of the Zippori cross section (Figure 3), and points 1-4 and 11 in the tree from Havat Hanania (Figure 2), might possibly have been caused by the 2.8mm diameter spanning numerous older unidentified rings, which would lead to higher concentrations of ^14^C, and in turn, an earlier date.

The following formula can be used to calculate the percentage of such assumed contamination (%_cont_):

(F^14^C_2009_ * (1-%_cont_)) + (F^14^C_cont_year_ * %_cont_) = F^14^C_measured_

**Equation 1.** Theoretical calculation of percent contamination of F^14^C from a certain year required for obtaining a given F^14^C value.

where F^14^C_2009_ is the fraction modern carbon (F^14^C) known for 2009, which is 1.0487 (the latest available from the latest bomb ^14^C calibration curve, NHZ2^32^). F^14^C_cont_year_ is the F^14^C value from the possible year of contamination, and the F^14^C_measured_ is the final result as measured by AMS.

The highest amount of atmospheric F^14^C occurred during 1963, with a value of nearly 2. This value has since been decreasing, and is expected to return to pre-bomb concentrations (below 1) in the near future, if it has not already. Thus, the worst case scenario would be that in which the tree had produced wood during 1963, and that this wood would be immediately adjacent to the most recent wood near the bark, causing contamination of the sample with higher levels of F^14^C, leading to an earlier date. This is the extreme scenario and does not take into consideration the more likely event, of wood having been produced between 1963 and the last year of growth, years in which the F^14^C was lower, which therefore would have diluted the signal. The following equations calculate the required percentage of contamination of old carbon, given the known F^14^C for each year of the “bomb peak”, which would result in the amount we have measured by AMS for the cellulose samples nearest the bark.

The F^14^C measured by AMS for point 9 from the outer circumference of the Havat Hanania cross section (Figure 2) was 1.20034. Rearranging Equation 1 and assigning F^14^C_2009_=1.0487 and F^14^C_measured_=1.20034 results in Equation 2a. For the Zippori cross section, using F^14^C_2009_=1.0487, and F^14^C_measured_ for point 9 as 1.21499, results in Equation 2b.

**(a)** %_cont_ = $\frac{0.15164}{{F14C}_{cont\_year}-1.0487}$ (**b)** %_cont_ = $\frac{0.16629}{{F14C}_{cont\_year}-1.0487}$

**Equation 2.** (a) Calculation for Hanania branch; (b) Calculation for Zippori whole tree

Constraining percent contamination to between 0 and 100, and F^14^C values to between one and two, which is the range of F^14^C between today and the increase from ~1955, the two versions of Equation 2 result in the following graphs:

Figure S 2. Percent contamination necessary of F^14^C in point sampled, in order to obtain 1.20034, a F^14^C value corresponding to 2009^32^. (a) Hanania, using equation 2a; (b) Zippori, using equation 2b.

From the above graphs, it is clear that the plausible range for obtaining a result of 1.20034 F^14^C would be between 100% input of 1.2 F^14^C (1986-carbon at the latest) with no 2009-carbon input at all, to 17% contamination of 1.9387 F^14^C (1963-carbon) and 83% 2009-carbon. Thus, even if the drilling method caused 10 or even 20 year old wood to be included along with the assumed 2009 ring, it could still not account for the observed F^14^C values. In addition, the tree from Havat Hanania was cut down alive in 2013, and thus the expected F^14^C value should be lower than 2009, requiring even more unidentified rings to have been accidentally sampled, making this scenario even less plausible.


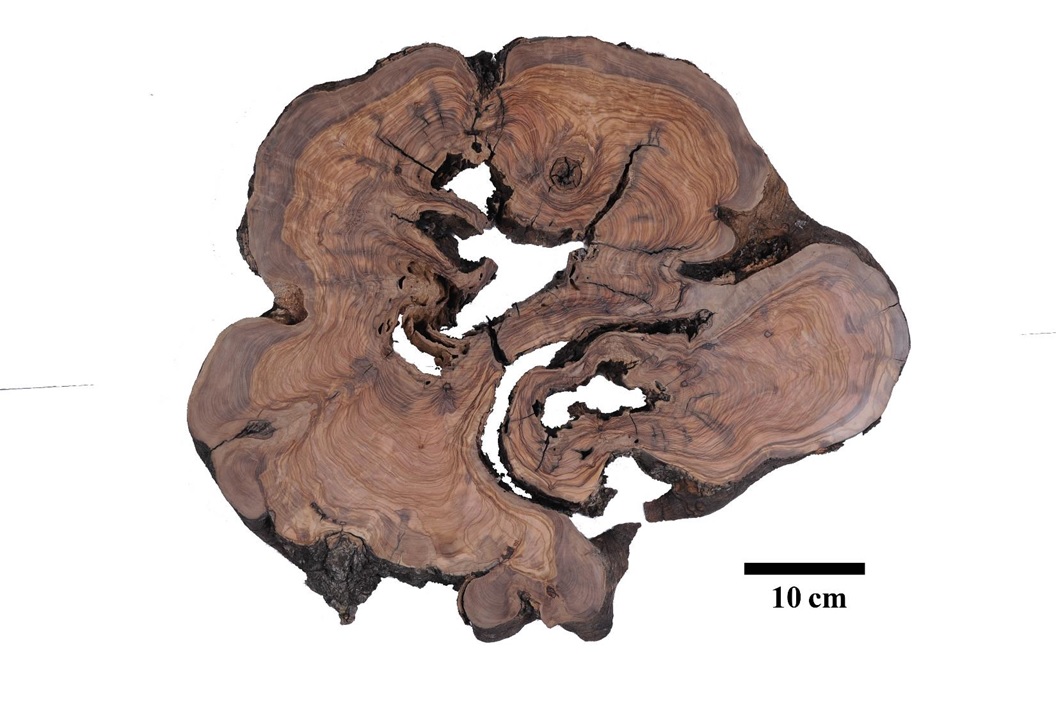


**Figure S 3.** Transverse section of olive tree trunk, cut down in 2013 in Zippori, northern Israel, several years after the tree had died. © Weizmann Institute of Science. Photo by: Itai Belson
